# Loss of functional MYO1C/myosin 1c, a motor protein involved in lipid raft trafficking, disrupts autophagosome-lysosome fusion

**DOI:** 10.4161/15548627.2014.984272

**Published:** 2015-01-28

**Authors:** Hemma Brandstaetter, Chieko Kishi-Itakura, David A Tumbarello, Dietmar J Manstein, Folma Buss

**Affiliations:** 1 Cambridge Institute for Medical Research; University of Cambridge; Cambridge, UK; 2 Institute for Biophysical Chemistry; Hannover Medical School; Hannover, Germany; 3 Center for Biological Sciences; University of Southampton; Highfield Campus; Southampton, UK

**Keywords:** autophagy, cholesterol, electron microscopy, lipid raft, lysosome, MYO1C, BafA_1_, bafilomycin A_1_, EM, electron microscopy, EGF, epidermal growth factor, EGFR, epidermal growth factor receptor, GFP, green fluorescent protein, KD, knockdown, LAMP1, lysosomal-associated membrane protein 1, LC3, microtubule-associated protein 1 light chain 3, MYO1C, myosin IC, MVB, multivesicular body, PB, phosphate buffer, PCIP, pentachloropseudilin, PtdIns(4, 5)P_2_, phosphatidylinositol 4, 5-bisphosphate, RFP, red fluorescent protein, RPE, retinal pigment epithelium

## Abstract

MYO1C, a single-headed class I myosin, associates with cholesterol-enriched lipid rafts and facilitates their recycling from intracellular compartments to the cell surface. Absence of functional MYO1C disturbs the cellular distribution of lipid rafts, causes the accumulation of cholesterol-enriched membranes in the perinuclear recycling compartment, and leads to enlargement of endolysosomal membranes. Several feeder pathways, including classical endocytosis but also the autophagy pathway, maintain the health of the cell by selective degradation of cargo through fusion with the lysosome. Here we show that loss of functional MYO1C leads to an increase in total cellular cholesterol and its disrupted subcellular distribution. We observe an accumulation of autophagic structures caused by a block in fusion with the lysosome and a defect in autophagic cargo degradation. Interestingly, the loss of MYO1C has no effect on degradation of endocytic cargo such as EGFR, illustrating that although the endolysosomal compartment is enlarged in size, it is functional, contains active hydrolases, and the correct pH. Our results highlight the importance of correct lipid composition in autophagosomes and lysosomes to enable them to fuse. Ablating MYO1C function causes abnormal cholesterol distribution, which has a major selective impact on the autophagy pathway.

## Introduction

Autophagy is a cellular degradation pathway to maintain cellular homeostasis in times of starvation and to eliminate long-lived or misfolded proteins, damaged organelles as well as cytosolic pathogens. In this process, cytoplasmic components are first sequestered within a specialized double-membrane vesicle termed the autophagosome. The autophagosome receives input from early and late endosomes in a stepwise maturation process to form an amphisome, which can then fuse with lysosomes to acquire the full set of hydrolases that enable content degradation.^1,2^

In mammalian cells, autophagosome-lysosome fusion depends on a variety of different factors including the small GTPase RAB7, the actin cytoskeleton as a tethering component, and also the correct lipid composition of the autophagosomal or lysosomal membrane.^3,4^ The importance of the correct lipid content was demonstrated through in vitro studies using isolated autophagosomes and lysosomes from animals challenged with a high-fat diet, which significantly changed the lipid composition of autophagosomes and lysosomes, thus leading to a reduction in their cholesterol content and a defect in autophagosome-lysosome fusion.^4^ However, not only lower levels of lysosomal cholesterol, but also the accumulation of this lipid in lysosomes, as seen in many different lysosomal storage diseases, reduces delivery of autophagic content to lysosomes.^5^

To further investigate the importance of cellular lipids for the regulation of autophagic activity, we analyzed whether changes in the cellular distribution of sphingolipid- and cholesterol-enriched lipid microdomains (herein referred to as lipid rafts) has an impact on efficient substrate degradation via autophagy. Our previous work identified MYO1C, a member of class I myosins, as a key regulator of trafficking of lipid rafts from intracellular storage compartments to the plasma membrane.^6^ MYO1C is a slow monomeric actin-based motor protein, adapted for sustained power mobility that links membrane cargo enriched in phospholipids, such as phosphatidylinositol 4,5-bisphosphate (PtdIns[4,5]P_2_), to the actin cytoskeleton. Membrane targeting of MYO1C involves a putative PH-domain present in the short C-terminal tail.^7,8^ This myosin motor is predominantly enriched in dynamic regions of the plasma membrane characterized by the presence of lamellipodia, filopodia, and membrane ruffles.^9^ In addition, MYO1C is recruited onto highly dynamic lipid-raft enriched membrane tubules in the endocytic recycling pathway, where it is required for delivery and exocytosis of lipid-raft enriched membranes at the plasma membrane.^6,10^ This function is supported by the finding that MYO1C facilitates the exocytosis and delivery of several raft-associated cargoes such as SLC2A4/GLUT4,^11,12^ AQP2/aquaporin 2,^13^ KIRREL/*NEPH1*^14^ as well as KDR/VEGFR2^15^ to the cell surface. At the cellular level, ablating MYO1C function results in a redistribution of lipid-raft enriched domains from the plasma membrane to intracellular compartments and an accumulation of these membranes in the perinuclear region.^6^ In addition, we also observed a very significant change in the morphology of late endocytic organelles in the form of enlarged swollen lysosomes following loss of MYO1C.^16^

Here, we demonstrate that changes in cellular cholesterol trafficking in MYO1C-depleted cells cause a significant increase in total intracellular cholesterol content. In cells missing functional MYO1C we observe a selective defect in autophagosome- and amphisome-lysosome fusion resulting in the accumulation of autophagic structures (phagophores, autophagosomes, and amphisomes), while the delivery and degradation of endogenous endocytic cargo such as the EGFR is not affected. Interestingly, although the loss of this myosin leads to morphological changes in endolysosomes, the enlarged organelles show no change in pH or in activity of lysosomal hydrolases. In summary, our results highlight the importance of MYO1C-dependent lipid trafficking for autophagosome-lysosome fusion, subsequent clearance of autophagic organelles, and degradation of mutant HTT/huntingtin protein aggregates via the autophagy pathway.

## Results

### Loss of MYO1C causes accumulation of autophagosomes

**Figure 1:**
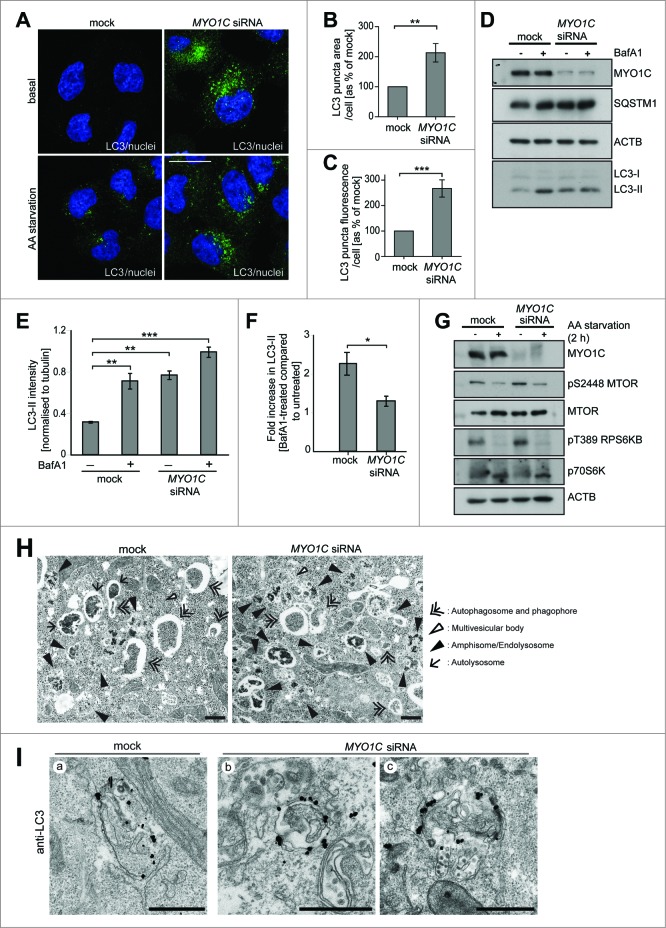
(**See previous page**). MYO1C depletion by siRNA causes accumulation of LC3-positive autophagosomes. (**A**) HeLa cells mock transfected or transfected with siRNA specific to *MYO1C* were either cultured under normal growth conditions or amino acid starved for 2 h, and were labeled with antibodies to endogenous LC3 for immunofluorescence microscopy. Bars = 10 μm. (**B, C**) LC3-positive puncta were quantified by high-throughput microscopy. Automated imaging and analysis software was used to calculate the puncta area (**B**) and fluorescence (**C**) per cell. A significant increase in the LC3-puncta area and fluorescence was observed in *MYO1C* siRNA-treated cells as compared to control cells. A total number of >50 ,000 cells from 3 independent experiments were analyzed. (**D**) For immunoblot analysis, control and MYO1C-depleted HeLa cells were either left untreated or treated with 100 nM bafilomycin A_1_ (BafA1) for 2 h. A representative blot is shown. (**E**) Quantification of protein gel blots from 3 independent knockdown experiments revealed an increase in LC3-II intensity upon MYO1C knockdown. (**F**) To determine the effect of MYO1C depletion on autophagosome biogenesis, the fold increase in normalized LC3-II intensity from bafilomycin A_1_-treated to untreated samples was calculated. Graphs represent the means ± s.e.m. (**G**) Control and *MYO1C* siRNA-depleted HeLa cells were amino acid starved for 2 h prior to processing for western blot analysis of MTOR signaling. (**H**) HeLa cells were cultured for 2 h in starvation medium and analyzed by conventional electron microscopy. Arrowheads highlight the presence of different autophagic and endocytic organelles. Bar = 500 nm. (**I**) Mock-treated and MYO1C-knockdown cells were labeled for immuno-electron microscopy using anti-LC3 antibodies. Bar = 500 nm.

The loss of MYO1C function results in redistribution of marker proteins that are associated with sphingolipid- and cholesterol-enriched lipid rafts, from the plasma membrane to intracellular compartments.^6^ Since disturbance of the cellular lipid balance, distribution, and membrane composition have a strong impact on the autophagy pathway,^4,5^ we investigated whether the depletion of functional MYO1C, either by siRNA-mediated knockdown (KD) or using the MYO1 inhibitor pentachloropseudilin (PCIP),^16^ has an effect on autophagosome biogenesis or degradation. To assess whether MYO1C is required for autophagy progression, we quantified the number of LC3-positive autophagosomes under basal conditions by immunofluorescence microscopy in MYO1C KD cells (**Fig. 1A**–**C**). Quantifying either the levels of endogenous LC3 (**Fig. 1A**) or using a stable cell line expressing GFP-tagged LC3 (**Fig. S1A**), we observed an accumulation of autophagosomes visualized by a 2-fold increase in the LC3-signal measured by LC3-area/cell or total LC3-fluorescence/cell (**Fig. 1B and C****; Fig. S1B, C, and D**). The accumulation of autophagosomes in MYO1C KD cells was confirmed by measuring the amount of lipidated LC3-II in the presence or absence of the vATPase inhibitor bafilomycin A_1_ (BafA_1_) by immunoblotting. BafA_1_ inhibits the acidification of endocytic structures and thereby blocks autophagosome-lysosome fusion. Under steady state conditions, in the absence of BafA_1_, MYO1C KD cells showed at least a 2-fold increase in the amount of LC3-II and an accumulation of the autophagy cargo SQSTM1/p62 as compared to mock-treated cells (**Fig. 1 D and E****; Fig. S1E**). The higher LC3-II levels in the absence of MYO1C were not linked to an increase in autophagosome biogenesis, since there was no increase in the accumulation of LC3-II in the presence of BafA_1_ in KD cells as compared to mock-treated cells (**Fig. 1D, E and F**). In addition, we also analyzed the effect of MYO1C protein depletion on MTOR signaling and autophagy activation in response to amino-acid starvation. Our results clearly show inactivation of MTOR signaling exhibited by the suppression of phosphorylation of MTOR and its downstream target, RPS6KB/p70S6K, in response to starvation in both mock- and MYO1C-depleted cells (**Fig. 1G**).

To further analyze the autophagy defect in MYO1C KD cells, we investigated the ultrastructural morphology of the accumulated LC3-positive organelles by conventional electron microscopy. Examples of the different autophagic structures, which can be classified according to their appearance as phagophores, autophagosomes, amphisomes, and autolysosomes^3^ together with late endocytic multivesicular bodies (MVBs), are highlighted by arrowheads in **Fig. 1H**. The ultrastructural analysis showed an increase in the number of amphisome/endolysosome-like organelles in MYO1C-depleted cells compared to mock-transfected cells (**Fig. 1H**). To confirm the identity of these organelles, control and MYO1C KD cells were labeled with antibodies to LC3 followed by silver-enhanced immunogold electron microscopy (**Fig. 1I**). Whereas LC3-positive autophagosomes were present in both control and MYO1C-depleted cells (**Fig. 1I, a and b**), LC3-positive amphisomes, which are a fusion product of LC3-positive autophagosomes and MVBs with numerous intralumenal vesicles (**Fig. 1I, c**), were more abundant in MYO1C KD cells. These results suggest a block late in the autophagy pathway and a potential accumulation of autophagic structures due to a defect in fusion with the lysosome.

**Figure 2:**
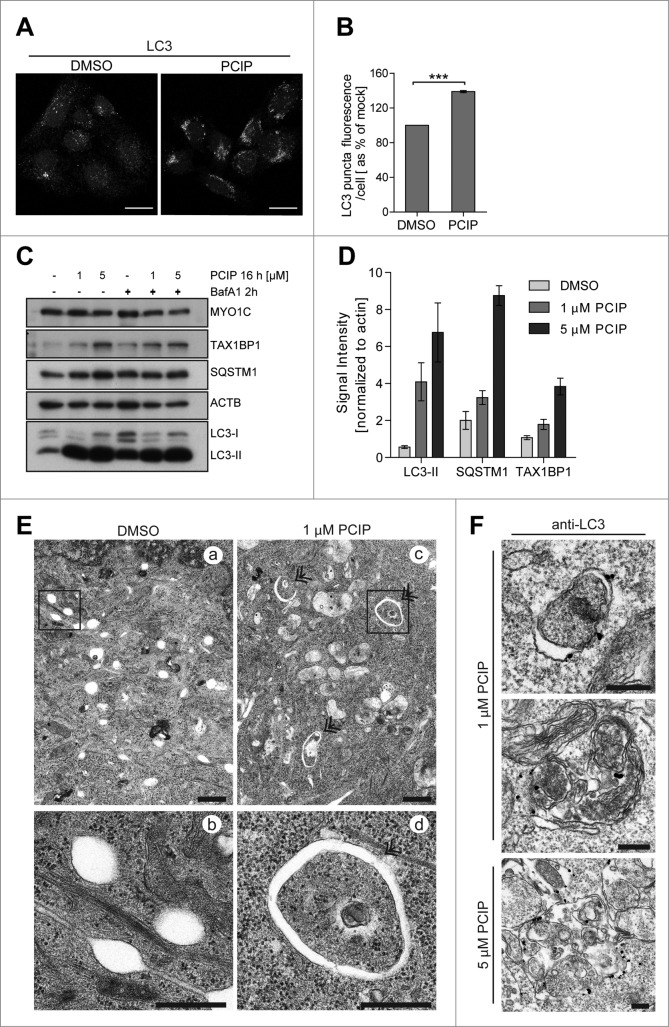
(**See previous page**). Inhibition of MYO1C function by the small molecule inhibitor PCIP causes accumulation of LC3-positive autophagosomes. (**A**) HeLa cells were incubated with 2 μM of the MYO1 inhibitor PCIP or equivalent amounts of DMSO for 16 h and processed for confocal microscopy using antibodies against endogenous LC3. (**B**) LC3-positive vesicles in control and PClP-treated cells were quantified by high-throughput microscopy. Automated imaging and analysis software revealed a significant increase in the LC3 puncta fluorescence per cell upon PClP treatment. A total number of >41 ,000 cells from 3 independent experiments, each performed in duplicate, were analyzed. (**C**) Western blot analysis of HeLa cell lysates treated with PCIP for 16 h in the absence or presence of 100 nM bafilomycin A_1_. (**D**) Quantification of LC3-II, SQSTM1, and TAX1BP1 protein expression from protein gel blots of PCIP-treated HeLa cells revealed a dose-dependent increase in LC3-II, SQSTM1, and TAX1BP1 levels. Results represent the mean (+/− s.d.) from >3 independent experiments. (**E**) Conventional electron microscopy of HeLa cells treated with 1 μM PCIP for 16 h. Panels (**b**) and (**d**) represent enlarged boxed regions in (**a**) and (**b**). Double arrows indicate autophagosomes and phagophores. Scale bar = 500 nm. (**F**) HeLa cells treated with PCIP for 16 h were labeled for immuno-electron microscopy using anti-LC3 antibodies. Scale bar = 500 nm.

The accumulation of LC3-positive autophagosomes, LC3-II, and the autophagy cargo receptors, SQSTM1 and TAX1BP1 (Tax1 [human T-cell leukemia virus type I] binding protein 1), was not only observed after siRNA depletion of MYO1C, but also when cells were treated with the small molecule inhibitor PCIP (**Fig. 2A, B, C and D**). This small molecule reduces the binding affinity of MYO1C for F-actin in the presence of nucleotide by binding in the cleft between the upper and lower 50-kDa region in the motor domain.^16^ Treatment with 1 or 5 μM PCIP for 16 h caused a dramatic increase in LC3-II (**Fig. 2C and D**); the accumulation of double-membrane autophagosomes under basal conditions was also observed by conventional electron microscopy (**Fig. 2E**). The nature of these autophagic structures was verified by immunolabeling with anti-LC3 (**Fig. 2F**). A significant increase in size of these LC3-positive organelles was observed in cells treated with 1 μM and even more in cells incubated with 5 μM PCIP (note the difference in scale bars in **Fig. 2F**). To further confirm that the autophagy defect was caused by the loss of MYO1C and was not due to off-target effects that were linked to the depletion of unrelated proteins, we performed KD experiments with the 4 individual siRNA oligonucleotides. The KD of MYO1C with each of the single siRNAs resulted in a similar accumulation of autophagosomes as seen by MYO1C depletion using SMARTpool siRNA or the inhibitor PCIP (**Fig. S2A**). Collectively, these results demonstrate that a functional MYO1C motor is required for autophagy progression under basal conditions, but is not required for autophagosome formation.

### Clearance of an autophagy-dependent cargo requires MYO1C

**Figure 3:**
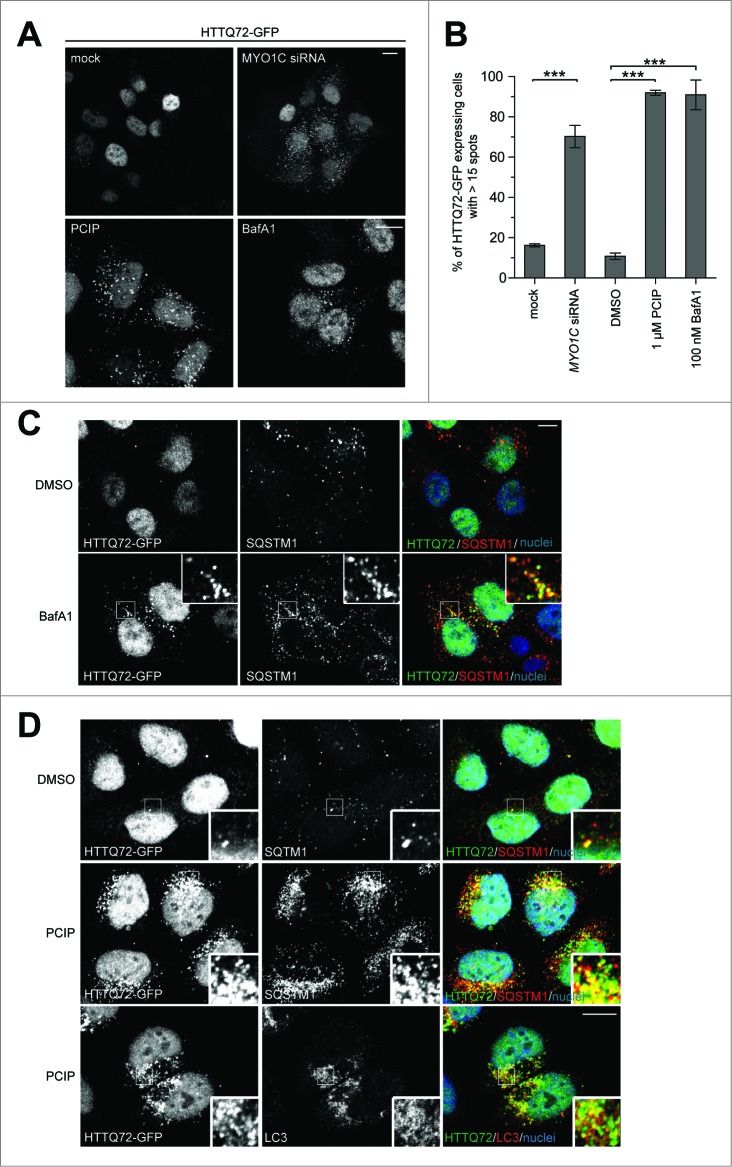
(**See previous page**). Protein aggregate clearance by selective autophagy requires MYO1C. (**A**) HeLa cells stably expressing HTTQ72-GFP were transfected with siRNA targeting *MYO1C* or treated with 1 μM PCIP, alongside appropriate controls, and labeled for immunofluorescence using antibodies to GFP. Bar = 10 μm. (**B**) HTTQ72-GFP aggregates were quantified in mock-, *MYO1C* siRNA-, DMSO-, 1 μM PCIP-, and 100 nM bafilomycin A_1_-treated cells. The results are represented as the percentage of HTTQ72-GFP-expressing cells with more than 15 GFP-positive spots per cell. MYO1C knockdown and PCIP treatment lead to a significant increase in the number of cells containing HTTQ72-GFP aggregates. Graphs represent the means ± s.e.m from 3 independent experiments. A total number of >3900 cells was analyzed. (**C**) HeLa cells stably expressing HTTQ72-GFP were treated with 100 nM bafilomycin A_1_ for 16 h prior to processing for immunofluorescence microscopy. Scale bar = 20 μm. (**D**) HeLa cells stably expressing HTTQ72-GFP were treated with 1 μM PCIP for 16 h prior to processing for immunofluorescence microscopy using antibodies to the indicated proteins. Nuclei in blue were labeled with Hoechst. Scale bar = 20 μm.

Autophagy may be a potential therapeutic approach for clearance of misfolded proteins caused by various pathologies, including Huntington's disease. Therefore, we assessed whether the MYO1C loss-of-function autophagy phenotype affects the autophagy-dependent clearance of HTT/huntingtin-polyQ protein aggregates.^17^ We analyzed whether loss of MYO1C expression has an effect on clearance of HTT protein containing a pathological poly Q expansion, which leads to misfolding and aggregation. We generated a HeLa cell line stably expressing a GFP-tagged HTT protein with a N-terminal 72 amino acid polyglutamine repeat (HTTQ72-GFP).^18^ In most cells this mutant form of HTT protein is cytosolic; only a few cells (less than 20%) contained multiple HTT aggregates (>15 /cell) (**Fig. 3A, B**). In contrast we observed a dramatic increase in the number of cells (> 70%) exhibiting multiple HTTQ72-GFP aggregates following MYO1C KD and PCIP treatment (**Fig. 3B**). The accumulation of HTTQ72-GFP aggregates and their colocalization with the autophagy receptor SQSTM1 and the autophagosome marker LC3, following MYO1C disruption, was consistent with the effects seen following inhibition of autophagosome-lysosome fusion using BafA_1_ (**Fig. 3A, B, C and D**). In summary, our results support a role for MYO1C in autophagy-dependent degradation of protein aggregates.

### MYO1C depletion leads to a defect in autophagosome-lysosome fusion

**Figure 4.**
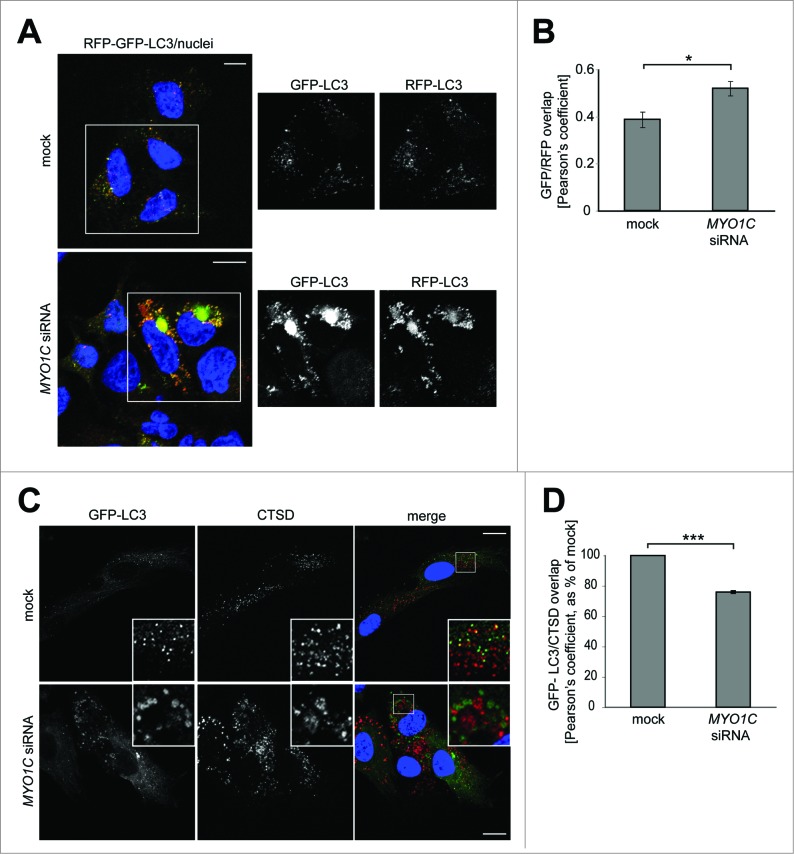
MYO1C depletion leads to defects in autophagosome-lysosome fusion. (**A**) HeLa cells stably expressing the RFP-GFP-LC3 reporter were either mock- or *MYO1C* siRNA-transfected and analyzed by confocal immunofluorescence. Single channel images of boxed regions are indicated to the right of merged color image. Cell nuclei are shown in blue. Bars = 10 μm. (**B**) The GFP/RFP signal overlap from confocal images of control and MYO1C knockdown cells expressing RFP-GFP-LC3 is represented as the Pearson's coefficient. A total number of >1000 cells from 3 independent experiments were analyzed. (**C**) Control and MYO1C siRNA-treated RPE cells stably expressing GFP–LC3 were stained with antibodies against GFP and CTSD for confocal microscopy. The inserts are enlarged representations of the boxed regions. Bars = 10 μm. (**D**) Quantification of GFP-LC3/CTSD signal overlap from confocal images of control and MYO1C knockdown RPE cells expressing GFP-LC3 is represented as the Pearson's coefficient. The decreased signal correlation in siRNA-treated cells confirms a role for MYO1C in autophagosome/lysosome fusion. Graph represents the means ± s.e.m.

Our data so far suggests that loss of functional MYO1C causes a block in fusion of autophagic organelles with lysosomes. To confirm this process in MYO1C KD cells, we used a pH-sensitive LC3-reporter construct containing both an RFP and GFP fusion tag at the N terminus.^19^ In this assay, the less acidic autophagosomes appear yellow due to red and green fluorescence; however, after fusion with a lysosome the drop in pH quenches the GFP-signal, and thus autolysosomes appear red. Using this approach, we found that in a stable HeLa cell line expressing the RFP-GFP-LC3 reporter, the loss of MYO1C causes an accumulation of yellow autophagosomes under basal conditions (**Fig. 4A**). Quantification following confocal microscopy revealed a significant increase in overlap of the GFP/RFP fluorescence signal in MYO1C KD cells compared to mock-transfected cells (**Fig. 4B**) indicating that MYO1C depletion causes impaired autophagosome-lysosome fusion. The LC3-positive autophagosomes and amphisomes in MYO1C-depleted cells showed less overlap in localization with CTSD/cathepsin D-positive lysosomes (labeled by the lysosomal protease CTSD) when compared to untreated control cells (**Fig. 4C and D**). This further confirms that loss of MYO1C causes a defect in fusion of autophagic organelles with lysosomes.

### Loss of MYO1C causes a dramatic change in lysosome morphology, but no change in the number of LAMP1-positive organelles

**Figure 5.**
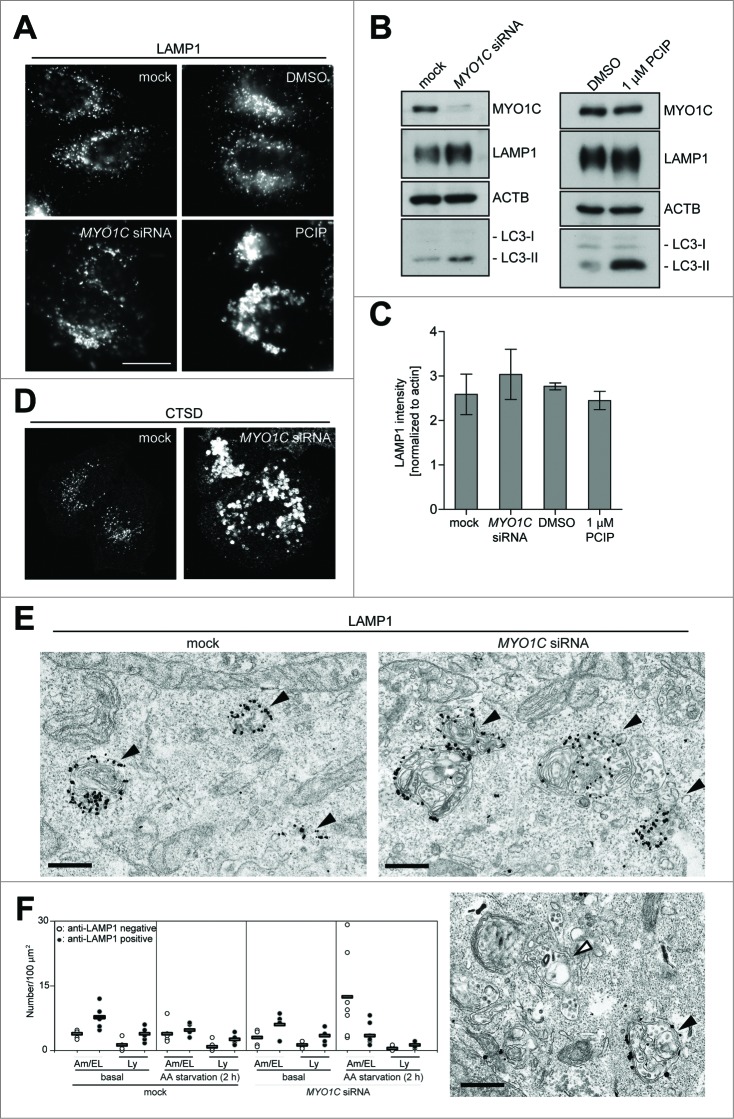
Effect of MYO1C knockdown on lysosome morphology. HeLa cells were either mock treated or transfected with *MYO1C* siRNA or incubated for 16 h with the MYO1 inhibitor PClP or equivalent amounts of DMSO as control before processing for confocal microscopy (**A, D**) or western blot analysis (**B**) using antibodies against the lysosomal marker proteins LAMP1 or the lysosomal protease CTSD. Bar = 10 μm. (**C**) Quantification of LAMP1 protein levels from protein gel blots following *MYO1C* siRNA depletion or PCIP treatment. Results represent the mean (+/− s.d.) from >3 independent experiments. (**E**) Mock-treated or *MYO1C* siRNA KD cells in complete medium were labeled for immuno-electron microscopy using anti-LAMP1 antibodies. Black arrowheads indicate LAMP1-positive amphisomes and the white arrowhead indicates a LAMP1-negative amphisome. Bar = 500 nm. (**F**) Graph showing the quantification of the number of LAMP1-positive or -negative lysosomes (Ly) or amphisomes (Am)/endolysosomes (EL) from at least 56 randomly selected areas, from each cell, for each condition in control or MYO1C KD cells growing in complete medium or under starvation conditions. Each data point indicates one cell.

To establish the cause of the inefficient delivery of autophagic material to the lysosome, we next investigated lysosome morphology and function in MYO1C-depleted cells. Our previous work demonstrated that ablating MYO1C function not only causes a dramatic cellular redistribution of cholesterol-enriched lipid raft domains,^6^ but also leads to a change in lysosomal morphology.^16^ Inhibition of MYO1C expression or activity using either a SMARTpool of *MYO1C*-specific siRNA (**Fig. 5A and D**), 4 single siRNA oligos (**Fig. S2B**) or the inhibitor PCIP (**Fig. 5A**) induced the formation of enlarged membrane structures, which contained the lysosomal hydrolase CTSD and the lysosomal membrane glycoprotein LAMP1. To further analyze the morphological changes in the late endocytic pathway, we performed pre-embedding immuno-electron microscopy of control and MYO1C KD cells with antibodies to LAMP1. The electron micrographs demonstrated that these enlarged LAMP1-positive structures (up to 2 μm in size) were morphologically similar to endolysosomes (the hybrid organelle resulting from late endosome-lysosome fusion),^20^ since they contained multilamellar membrane whirls that are typically seen in lysosomes, as well as different sizes of intralumenal vesicles often observed in late endosomes (**Fig. 5E****; Fig. S3C**). While evaluation of LAMP1 by western blot only indicated slight fluctuations in overall protein levels, quantification of LAMP1 by light microscopy showed an increase in LAMP1-immunoreactivity (**Fig. 5B and C****; Fig. S3A**), which was linked to the increase in size^21^ (**Fig. S3D**) and was not caused by an increase in the number of LAMP1-positive organelles, which stayed constant in the presence or absence of MYO1C (**Fig. S3B**).

This result was confirmed by quantitative immuno-electron microscopy, which also demonstrated no difference in the number of LAMP1-positive lysosomes or endolysosomes in control and MYO1C KD cells under steady-state conditions (**Fig. 5F**). However, after amino acid starvation, we observed a significant accumulation in the number of LAMP1-negative amphisomes in MYO1C KD cells (**Fig. 5F**; white arrowhead in **Fig. 5E**). Taken together our results so far suggest that loss of functional MYO1C increases the size but not the number of LAMP1- and CTSD-positive endolysosomes and leads to an accumulation in the number of LAMP1-negative amphisomes.

### The enlarged lysosomes in MYO1C-depeleted cells show no defect in lysosomal activity

**Figure 6:**
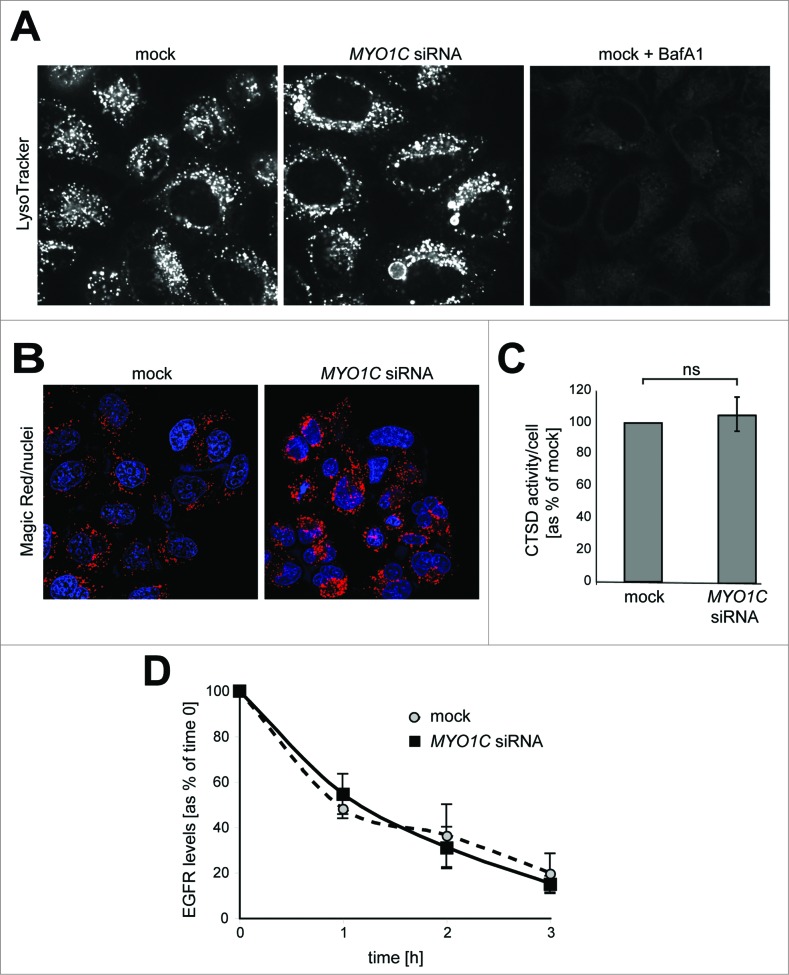
(**See previous page**). Analysis of lysosome function in MYO1C-depleted cells. (**A**) Mock, MYO1C-depleted cells, and bafilomycin A_1_-treated cells were incubated with the LysoTracker Red probe and imaged using live cell microscopy to monitor lysosomal pH. Swollen lysosomes in MYO1C knockdown cell appear to be acidic. (**B**) To determine lysosomal enzyme activity in live cells control and MYO1C-depleted HeLa cells were incubated with Magic Red. Confocal microscopy was used to image cathepsin–associated hydrolysis of Magic Red into its red fluorescent form. (**C**) Calculation of cathepsin activity using Volocity Imaging software revealed no significant difference between cathepsin activity in control and MYO1C-knockdown cells. Values are means ± s.e.m from 3 independent experiments (>1900 cells). ns, not significant. (**D**) To measure EGFR degradation in control and *MYO1C* siRNA-treated HeLa cells, cells were serum-starved overnight, and incubated with 100 μg/ml cycloheximide for 2 h before stimulating with 100 ng/ml EGF for 0, 1, 2 and 3 h. EGFR levels were quantified from immunoblots and normalized to a loading control. No significant difference in the rate of EGFR degradation was observed in control and knockdown cells. Values are means ± s.e.m from 3 independent knockdown experiments.

Enlarged late endocytic organelles are often observed in lysosomal storage disorders. In these diseases cholesterol and other lipids accumulate in lysosomes, which may cause defects in proteolytic activity or reduced ability of lysosomes to fuse with endosomes or autophagosomes.^22,23^ To establish whether the morphological changes associated with loss of MYO1C affect the homeostasis and physiological activity of the enlarged lysosomes, we first used the pH-sensitive dye LysoTracker Red to label acidic lysosomes in live cells. Mock transfected and MYO1C KD cells displayed very similar intensity in LysoTracker Red staining, indicating no major change in lysosomal acidity after loss of MYO1C (**Fig. 6A**). After treatment with BafA_1_, which inhibits acidification of endosomes and lysosomes, we observed a complete loss of LysoTracker Red signal (**Fig. 6A**). We next determined whether these swollen lysosomes still perform their degradative functions by analyzing proteolytic activity of lysosomal cathepsin in MYO1C-depleted cells. We measured intracellular cathepsin activity directly in lysosomes using an artificial commercial cathepsin-substrate peptide (Magic Red ^TM^), which is linked to the fluorophore cresyl violet and emits a red fluorescence signal at 550–590 nm after enzymatic cleavage. Using this artificial substrate, we observed *in situ* no difference in cathepsin-activity, measured as the amount of red fluorescent signal, between mock or MYO1C KD cells (**Fig. 6B and C**). Finally, we used EGFR (epidermal growth factor receptor) as an endogenous substrate to monitor lysosomal activity. EGFR is a typical member of the receptor tyrosine kinase family, which after ligand binding-induced activation is endocytosed and delivered to lysosomes for degradation. Following stimulation of control and MYO1C KD cells with EGF for 1, 2 or 3 h to induce internalization and trafficking of the receptor to lysosomes, the rate of EGFR degradation was assessed by immunoblotting. Consistent with our results from the cathepsin activity assay, no defect in ligand-induced EGFR degradation was observed after loss of functional MYO1C (**Fig. 6D**). Taken together, these data demonstrate that, although absence of MYO1C causes dramatic changes in endolysosomal morphology, this has no effect on lysosomal function and degradation of endocytic cargo.

### MYO1C KD cells display defects in intracellular cholesterol distribution

**Figure 7:**
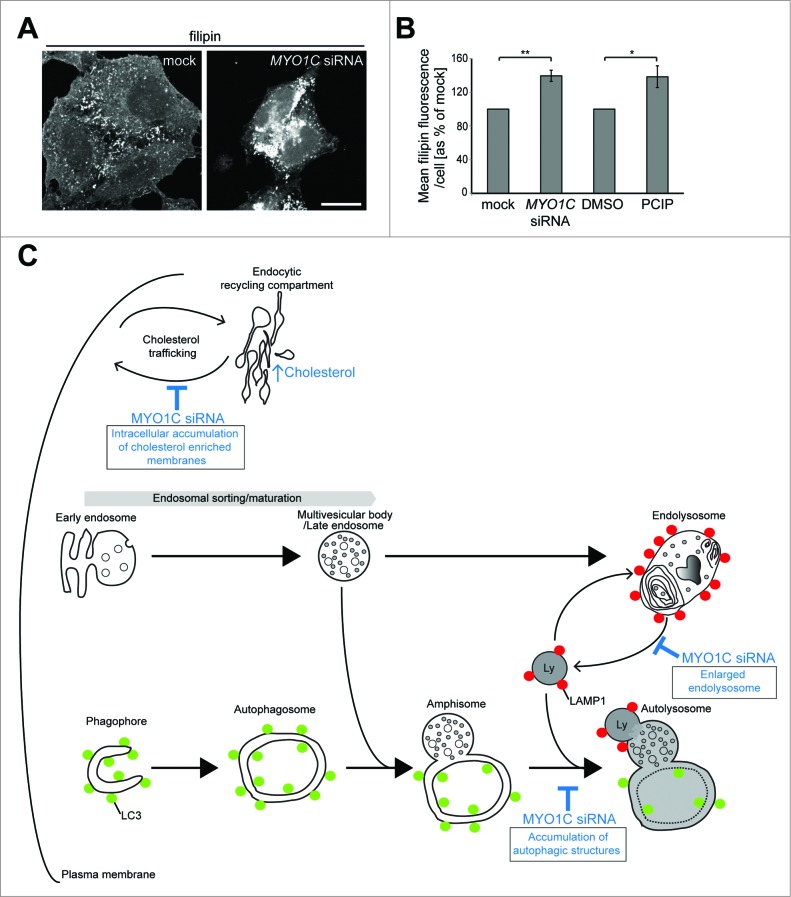
(**See previous page**). MYO1C-depleted cells contain more total cellular cholesterol, trapped in intracellular storage compartments. (**A**) Confocal z-projection microscopy images of cellular cholesterol, imaged with filipin, from mock and *MYO1C* siRNA-depleted HeLa cells. Scale bar = 20 μm (**B**) Total cholesterol levels in mock and MYO1C-knockdown cells and DMSO- and PClP-treated cells were quantified by high-throughput microscopy. Automated imaging and analysis software was used to calculate the total filipin fluorescence per cell. Loss of MYO1C caused a significant increase in cholesterol as compared to control cells. In total >149 ,000 cells from 3 independent experiments, each performed in triplicate, were analyzed. Graphs represent the means ± s.e.m. (**C**) Model of the autophagy and endocytic pathway highlighting defects (shown in blue) observed in MYO1C-depleted cells. Loss of functional MYO1C causes a defect in lipid raft recycling from the perinuclear recycling compartment back to the cell surface. This leads to intracellular accumulation of cholesterol-enriched membranes.^6^ In the classical endocytic pathway, incoming cargo first moves through early endosomes, which then acquire an increasing number of intralumenal vesicles and mature into late endosomes (LE)/multivesicular bodies (MVB). The fusion of a LE/MVB with a lysosome generates a transient hybrid organelle, the endolysosome, in which content degradation can take place. In MYO1C-depleted cells, we observe the accumulation of enlarged LAMP1- and CTSD-positive endolysosomes. In the autophagy pathway, cytosolic material is sequestered by expansion and closure of a phagophore, forming double-membrane vesicles called autophagosomes. These autophagosomes mature by fusion with early and late endosomes to form an intermediate organelle called the amphisome, which fuses with lysosomes to enable content degradation. Ablating MYO1C activity leads to an accumulation in the number of autophagic structures suggesting a block in fusion of autophagic organelles with lysosomes.

Since ablating MYO1C expression causes the redistribution of lipid rafts from the plasma membrane to an intracellular compartment, we next investigated whether there was an abnormal distribution of cholesterol-enriched membranes in MYO1C KD cells. A similar phenotype can be observed in lysosomal storage diseases, such as Niemann-Pick disease type C, which is linked to an increase of cellular cholesterol and glycolipids concentrated in swollen lysosomes. To measure cellular cholesterol levels and visualize cholesterol-positive content in cells, we used the fluorescent dye filipin, which stains unesterified cholesterol *in situ* and thereby illuminates intracellular cholesterol storage organelles. We evaluated filipin fluorescence by confocal microscopy and quantified total filipin fluorescence in mock-transfected and MYO1C KD cells as well as in DMSO-treated or PCIP-treated cells using automated microscopy (**Fig. 7A and B**). The results shown in **Figures 7A and B** clearly demonstrate that, similar to Niemann-Pick disease type C, the overall cellular cholesterol levels were significantly increased in MYO1C-depleted cells. In control cells, filipin-staining was present on the cell surface and on intracellular organelles, which showed some overlap with LAMP1 (**Fig. 7A****; Fig. S4**). In MYO1C KD cells, however, most of the filipin staining was redistributed from the plasma membrane to intracellular compartments, but did not exhibit a preferential accumulation in a LAMP1-positive compartment (**Fig. 7A****; Fig. S4**). These results suggest that loss of MYO1C function causes intracellular mistrafficking of cholesterol-enriched membranes, thus resulting in an accumulation and aberrant distribution of cholesterol.

## Discussion

Here we report that loss of MYO1C function specifically inhibits clearance of autophagosomes and sequestered protein aggregates, but does not impair degradation of endocytic cargo. After loss of functional MYO1C, we observe a set of distinct phenotypes: the accumulation of autophagic organelles, suggesting a block in autophagy progression, and the formation of swollen LAMP1 as well as CTSD-positive endolysosomes (summarized in our model shown in **Fig. 7C**). In addition, our previous work demonstrated that MYO1C-depleted cells show redistribution of cholesterol and sphingolipid-enriched lipid rafts from the plasma membrane and accumulation of these lipids in intracellular membrane compartments.^6^ This defect in intracellular cholesterol trafficking leads to an accumulation of total cellular cholesterol (**Fig. 7**).

The reduction in autophagosome degradation could be caused by either (1) a defect in autophagosome maturation, (2) reduced proteolytic activity of the lysosomal enzymes or (3) impaired autophagosome fusion with the lysosome. We investigated these different possibilities and found no obvious defect in delivery and degradation of endocytic cargo in lysosomes; the endocytic pathway required during autophagosome maturation is fully functional, since we observed the accumulation of amphisomes, which are hybrid organelles composed of late endosomes/MVBs and autophagosomes, in MYO1C-knockdown cells. Furthermore, no changes in lysosomal pH or reduction in proteolytic activity were observed when measuring cathepsin activity using a reporter substrate or when analyzing the degradation of endogenous endocytic reporter molecules such as EGFR. These results demonstrate that loss of MYO1C does not impair endolysosomal or lysosomal activity. This leaves us with the third possibility, a reduction in fusion of autophagosomes with the late endocytic proteolytic compartment, which would explain the defect in autophagic cargo degradation and the observed increase in the number of these amphisome-like organelles.

The absence of functional MYO1C leads to the formation of enlarged late endocytic and/or lysosomal structures, which contain LAMP1 as well as CTSD. Ultrastructural analysis highlights the presence of lysosomal membrane whirls as well as large numbers of intralumenal vesicles; these are morphological characteristics typically found in endolysosomes, the hybrid organelle formed by late endosome/MVB fusion with a lysosome, which provides the degradative enzymes to make the hybrid organelles competent for proteolysis.^20,24^ It is thought that membranes are retrieved from this hybrid organelle by vesicle trafficking to counterbalance the constant membrane influx from the endocytic pathway. This may also provide a pathway for the reformation of the classical dense core lysosome, which is the storage compartment for lysosomal hydrolases.^20^ It has been suggested that PtdIns(4,5)P_2_ regulates lysosome reformation following autophagy activation.^25,26^ Since MYO1C contains a PtdIns(4,5)P_2_-specific PH domain, we evaluated whether loss of MYO1C affects PtdIns(4,5)P_2_ on the plasma membrane using a GFP-PLCD/PLCδ-PH probe. Our results indicate no apparent difference in PtdIns(4,5)P_2_ on the plasma membrane in MYO1C KD or PCIP-treated cells (**Fig. S5**).

The accumulation of these swollen endolysosomes in MYO1C KD and PCIP-treated cells may be linked to abnormal cholesterol levels in this LAMP1-positive compartment, which can change membrane properties and may reduce membrane retrieval from this organelle. Alternatively, MYO1C might have a direct function in membrane budding from endolysosomes, similar to its postulated role in cargo sorting and vesicle/tubule formation at the perinuclear recycling compartment.^27^ Although we are not able to detect significant amounts of MYO1C on LAMP1-positive organelles under steady-state conditions, previous reports show that overexpression of VPS18, a component of the mammalian HOPS complex, causes clustering of late endosomes and/or lysosomes in actin-rich areas and recruitment of MYO1C to these compartments.^28^ These results suggest that MYO1C may indeed be able to associate with endolysosomes, however, under steady-state conditions the recruitment of a limited number of MYO1C motors from the cytosolic pool might be transient and therefore difficult to detect by standard immunofluorescence microscopy.

In a number of lysosomal storage diseases, including Niemann-Pick type C disease, defects in cholesterol and lipid trafficking lead to accumulation of lipids in late endocytic compartments, which results in a strong inhibitory effect on autophagosome clearance.^23,29,30^ Our results are consistent with previous studies that demonstrated a dramatic reduction in autophagosome-lysosome fusion *in vitro*, when levels of cholesterol in the membrane of autophagosomes or lysosomes were changed either by chemical treatment of isolated organelles or by feeding animals a high-fat diet before isolating the organelles for *in vitro* fusion assays.^4^ Although the molecular mechanism of how lipid content regulates autophagosome-lysosome fusion remains to be determined, it is likely that changes in cholesterol levels have an impact on membrane rigidity or fluidity. This will change the physical parameters of the lipid bilayer surrounding the autophagosome, endolysosome, and lysosome and thus affect the lateral dynamics and availability of membrane proteins required for the fusion process.

Interestingly, although close links exist between endocytosis and autophagy and many of the regulatory factors and machinery are shared between the 2 pathways,^31^ loss of MYO1C and the resulting changes in lipid composition selectively inhibit autophagosome-lysosome fusion, but do not inhibit delivery, fusion, and degradation of endocytic cargo in the endolysosome or lysosome. Therefore, the data presented in this work support the model whereby fusion of autophagosomes with lysosomes utilizes a specific mechanism that is distinct from other fusion events in the endocytic pathway. Differences between the mechanism of autophagosomal-lysosomal and endosomal-lysosomal fusion is also highlighted by experiments using the drug thapsigargin, which selectively inhibits autophagosome-lysosome fusion, while having no effect on the degradation of endocytic cargo. ^32^

In summary, this work clearly highlights that different myosin motor proteins have very distinct functions along the autophagy pathway. Our previous work demonstrated that MYO6/myosin VI and its adaptor protein TOM1 are required for delivery of early endosomes to the autophagosome,^18^ a process recognized to be important during autophagosome maturation.^33,34^ In contrast, MYO1C regulates intracellular trafficking of cholesterol-enriched membranes and deletion of this motor may cause a change in the lipid composition of autophagic and late endocytic organelles, which has a clear impact on the fusion efficiency between these compartments. In addition, the separate functions of MYO1C and MYO6 in the autophagy pathway may be due to their different motor properties; MYO6 although also a slow myosin, exists as a monomer or dimer, and moves toward the minus end of actin filaments, whereas MYO1C is a slow monomeric myosin, which moves toward the plus end of actin filaments. Thus in the cell these 2 motors might perform distinct functions required for the operation of the autophagy pathway.

## Materials and Methods

### Antibodies

The following commercial antibodies were used: rabbit polyclonal antibodies to GFP (Invitrogen, A11122), MYO1C (Sigma-Aldrich, HPA001768), EGFR (Santa Cruz Biotechnology, 1005 sc-03); mouse monoclonal antibodies to GFP (Abcam, ab 1218), LC3 (MBL International, M152–3), SQSTM1 (BD Biosciences, 610832), LAMP1 (Developmental Studies Hybridoma Bank, University of Iowa, H4A3-c), TUBA4A/tubulin (Sigma-Aldrich, T9026), CTSD/cathepsinD (BD Biosciences, 610800), FLOT1/flotillin 1 (BD Biosciences, 610820), FLOT2/flotillin2 (BD Biosciences, 610383), phospho-Thr389 RPS6KB/p70S6 kinase (Cell Signaling Technology, 9206), RPS6KB/p70S6 kinase (Cell Signaling Technology, 9202), phospho-Ser2448 MTOR (Cell Signaling Technology, 2971), MTOR (Cell Signaling Technology, 2972). Alexa Fluor 488- and 568-conjugated secondary antibodies were from Invitrogen (A21429 and A11029).

### Cell culture, siRNA transfection, and drug treatment

HeLaM cells^35^ were cultured in RPMI 1640 medium (Sigma, R8758) and retinal pigment epithelium (RPE) cells in 50:50 DMEM:F12 Ham medium (Sigma, D6429 and N4888) supplemented with 30 mM sodium bicarbonate, all containing 10% fetal calf serum, 2 mM L-glutamine, 100 U/ml penicillin and 100 μg/ml streptomycin in a 5% humidified atmosphere. As described and characterized previously^18^ RPE cells stably expressing GFP-LC3 and HeLa RFP-GFP-LC3 cell lines were created using pIRESneo2 plasmids, selected in medium containing 500 μg/ml G418 (Gibco, 10131–027); and the HTTQ72-GFP stable HeLa cells line was generated using the Lent-X Lentiviral expression system (Clontech, 631247). The GFP-PLCD-PH stable expressing HeLa cells were generated using the GFP-C1-PLCD/PLCdelta-PH plasmid (Addgene, 21179) developed by Tobias Meyer (Stauffer, 1998), following selection in G418.

All siRNA oligos (ON-TARGETplus single oligos and SMARTpool) were obtained from Dharmacon. For successful protein depletion, cells were transfected twice with siRNA on d 1 and 3 using OligofectAMINE (Invitrogen, 12252–011). On d 5 cells were processed for the indicated assays. Protein knockdown efficiency was assessed by immunoblotting.

Bafilomycin A_1_ (used at a final concentration of 100 nM, 2 h) was purchased from Sigma-Aldrich (B1793), and the MG132 inhibitor (used at 1 μM for 2 h) from Millipore (CAS 133407–82–6). Cells were incubated with 3 μM U18666A drug (Millipore, 662015) for 16 h. The MYO1C inhibitor PClP, a kind gift of H.-J. Knölcker (Technical University of Dresden), was used at a concentration of 1–5 μM in DMSO for 16 h, as previously described.^21^

### EGFR degradation assay

Control and knockdown HeLa cells were serum starved overnight, incubated with 100 μg/ml cycloheximide (Sigma, C4859) for 2 h before stimulation with 100 ng/ml EGF (Sigma, E9644). Cells were lysed at the indicated time points and lysates were blotted as described below.

### Immunoblotting

Cells were washed in PBS (Sigma, D8537) and lysed in 6 M urea-SDS loading buffer (2% SDS, 30% glycerol, 1 M β-mercaptoethanol, 6 M urea, 0.125 M Tris, pH 6.8, 0.01% bromophenol blue), and separated by SDS-PAGE followed by immunoblot analysis with the indicated antibodies. Western blots were developed using the ECL detection reagents (GE Healthcare, RPN2106V1 and V2). Quantitative immunoblotting was performed following protein transfer to ImmobilonFL PVDF membranes (Millipore, IPFL00010) and incubation with Alexa Fluor 680-conjugated secondary antibodies (Invitrogen, 926–32212D and 926–32213D), before scanning on a LI-COR Odyssey Imaging system (LI-COR Biosciences). Following background correction, the integrated intensity of proteins of interest bands were normalized to a loading control before quantification.

### Immunofluorescence microscopy

Cells plated on coverslips were fixed with 4% paraformaldehyde (or cold methanol for CTSD labeling), permeabilized with 0.2% Triton X-100 (BDH, 306324N) and quenched with 10 mM glycine. To reduce cytosolic background cells were extracted with 0.02% saponin (Sigma, F47036) for 30 sec before fixation. Fixed cells were blocked with 1% BSA (Fisher Scientific, BP1605–100) in PBS and processed for indirect immunofluorescence using the indicated primary antibodies followed by Alexa Fluor 488- or Alexa Fluor 568-coupled secondary antibodies. F-actin was visualized using Alexa Fluor 568-coupled phalloidin (Sigma Aldrich, A12380) and cell nuclei were stained with Hoechst (Invitrogen, H3570). To visualize cholesterol, nonpermeabilized cells were labeled with freshly prepared filipin-complex (Sigma, F4767) in DMSO at a final concentration of 25 μg/ml. The MagicRed CathepsinB Assay Kit (ImmunoChemistry Technologies, 937) was used following the manufacturer's instructions.

Images were obtained at a magnification of 63x using a Zeiss LSM710 confocal microscope or a Zeiss Axioplan epifluorescence (Zeiss), equipped with a Hamamatsu Orca R2 camera (Hamamatsu Photonics). To analyze colocalization, the Pearson's coefficient was calculated from confocal images using the Volocity 5.2 software (Perkin Elmer).

### Automated quantification

ArrayScan VTi 6.6.1.4 HighContentScreening microscope and software (Cellomics) were used for the automated quantification of lysosome number/cell, LAMP1 intensity, total cholesterol levels/cell and autophagosome size. Cells were either seeded into 96-well view plates (Perkin Elmer, 6005182) or on glass coverslips, and processed for immunofluorescence as described above using the indicated antibodies and Hoechst stain to identify each individual cell. Images were captured using a 20× or 40× lens and analysis was performed with the Cellomics Spot Detector V4 or Target activation V4 algorithm applications.

### Electron microscopy

HeLa cells were cultured on collagen-coated plastic coverslips (Sumitomo Bakelite, MS-0113 KZ) and fixed in 2.5% glutaraldehyde in 0.1 M sodium phosphate buffer, pH 7.4 (phosphate buffer, PB) for 2 h. The cells were then washed 3 times in PB, before post-fixing for 1 h in 1% osmium tetroxide in 0.1 M PB. The fixed cells were then dehydrated and embedded in Agar 100 resin (Agar Scientific, R1030) according to a standard protocol.^36^ Ultrathin sections were stained with uranyl acetate and lead citrate and observed under a FEI Tecnai Spirit electron microscope (FEI, Hillsboro, Oregon, USA).

For immunoelectron microscopy analysis of endogenous LC3 and LAMP1, cells were fixed with 4% paraformaldehyde in 0.1 M PB for 2 h on ice. After washing 3 times with PB, the cells were permeabilized in liquid nitrogen, before blocking with 0.005% saponin, 10% BSA, 10% normal goat serum (Sigma, G9023), and 0.1% cold water fish skin gelatin (Sigma, G7765) in PB.^37^ Cells were incubated with mouse monoclonal anti-LC3 (Cosmo Bio, CTB-LC3–2–1C; diluted 10×) or mouse monoclonal anti-LAMP1 (diluted 250×) in blocking solution overnight. The cells were then washed in PB containing 0.005% saponin several times and incubated for 2 h with goat anti-mouse IgG conjugated to colloidal gold (1.4 nm diameter; Nanoprobes, 2002). Cells were washed with PB and fixed with 1% glutaraldehyde in PB for 10 min. After washing, the gold labeling was intensified (6 min at room temperature) by using a gold enhancement kit (Nanoprobes, 2113). After washing in distilled water, cells were postfixed in 1% OsO_4_ containing potassium ferrocyanide for 60 min at 4°C, washed in distilled water, incubated with 30% ethanol for 10 min, 50% ethanol 10 min and stained with 2% uranyl acetate in 70% ethanol for 2 h. The cells were further dehydrated with a graded series of ethanol and were embedded in Agar 100 resin. Ultrathin sections were double stained with uranyl acetate. ^37^

### Statistical significance

Comparisons between 2 data sets were performed using the Student *t* test, between 3 or more data points using ANOVA combined with post-hoc Bonferroni's multiple comparison test (GraphPad Prism 5.01).

## Supplementary Material

2013AUTO0738R4_Supplemental_Figures_and_Legends.zip
