# Supplementary material for: Loss of functional MYO1C/myosin 1c, a motor protein involved in lipid raft trafficking, disrupts autophagosome-lysosome fusion
Source: Autophagy. 2015 Jan 28;10(12):2310–23. doi: 10.4161/15548627.2014.984272 (PMC4502697; doi:10.4161/15548627.2014.984272)
Supplement: 2013AUTO0738R4_Supplemental_Figures_and_Legends.zip [file kaup-10-12-984272-s001.zip › Supplemental Figure Legends.pdf]

Figure S1. siRNA depletion of MYO1C causes an accumulation of autophagosomes. (**A**) HeLa cells stably expressing GFP-LC3 were MYO1C depleted with siRNA and cultured in growth media or amino acid starved for 2 h prior to processing for immunofluorescence microscopy. Nuclei in blue were labeled with Hoechst. Scale bar = 20 μm. LC3-positive puncta were quantified by high-throughput microscopy. Automated imaging and analysis software was used to calculate the puncta area (**B**) and fluorescence (**C**) per cell. (**D**) RPE cells stably expressing GFP-LC3 were MYO1C depleted with siRNA followed by processing for immunofluorescence microscopy. To reduce the cytosolic background the cells were extracted with saponin prior to fixation. As shown, there is an increase in GFP-LC3 puncta in MYO1C-depleted cells. (**E**) Following *MYO1C* siRNA depletion in HeLa cells, quantification of SQSTM1 protein expression from western blots revealed an increase in SQSTM1 levels following *MYO1C* siRNA KD. Results represent the mean (+,- s.d.) from >3 independent experiments. *p>0.05.

Figure S2. Single siRNA oligonucleotides specific to *MYO1C* cause accumulation of autophagosomes and swelling of lysosomes. HeLa cells were either mock transfected or treated with single siRNA oligos targeting *MYO1C* and (**A**) labeled with antibodies to endogenous LC3 or (**B**) LAMP1 for confocal immunofluorescence microscopy. Bars = 10 μm. (**C**) HeLa cells were either mock transfected, treated with single siRNA oligos to *MYO1C* or with a siRNA SMARTpool combining all 4 oligos. Cell lysates were prepared and were immunoblotted with antibodies against MYO1C and TUBA/α-tubulin as a loading control to confirm successful MYO1C protein KD.

Figure S3. Quantification of morphological changes in LAMP1-positive organelles. To quantify swelling of lysosomes, mock or *MYO1C* siRNA transfected cells were labeled with antibodies to LAMP1 for high-throughput microscopy. Automated imaging software was used to quantify the total LAMP1 fluorescence intensity (**A**) and the number of lysosomes (**B**) per single cells. A total number of >7700 cells from 3 independent experiments, each performed in triplicate, were analyzed. Values are means ± s.e.m. ns = non significant. (**C**) Immunogold labeled electron microscopy against LAMP1 of PCIP-treated HeLa cells. Panels (**b**) and (**d**) represent enlargements of the boxed regions in panels (**a**) and (**c**). Scale bar = 500 nm (**a, c**) 100 nm (**b, d**). (**D**) Quantification of LAMP1-positive endolysosome size by electron microscopy in PCIP-

treated HeLa cells. Values were calculated as the average diameter of the long and short axis of each LAMP1-positive endolysosome organelle. The results represent the average from >20 organelles from 4 independent cells for each group, ± s.e.m.

Figure S4. Accumulation of intracellular cholesterol in MYO1C KD cells, with only a limited accumulation in a LAMP1-positive compartment. HeLa cells, either mock or *MYO1C* siRNA transfected, were processed for confocal microscopy and stained with filipin (red) and immunolabelled for LAMP1 (green). Images represent z-projections and illustrate an accumulation of filipin staining in MYO1C KD cells, with only a limited proportion colocalizing with LAMP1. Scale bar = 20 μm.

Figure S5. Loss of MYO1C has no effect on PtdIns(4,5)P_2 at the plasma membrane. HeLa cells stably expressing GFP-PLCD-PH were transfected with *MYO1C* siRNA or incubated with 1 μM PCIP for 16 h, followed by processing for immunofluorescence microscopy. Nuclei in blue were labeled with Hoechst. Scale bar = 20 μm. GFP-PLCD-PH is enriched on the plasma membrane in both control cells and in cells with ablated MYO1C function.
