# Supplementary material for: Loss of functional MYO1C/myosin 1c, a motor protein involved in lipid raft trafficking, disrupts autophagosome-lysosome fusion
Source: Autophagy. 2015 Jan 28;10(12):2310–23. doi: 10.4161/15548627.2014.984272 (PMC4502697; doi:10.4161/15548627.2014.984272)
Supplement: 2013AUTO0738R4_Supplemental_Figures_and_Legends.zip [file S3.pdf › S3.pdf]

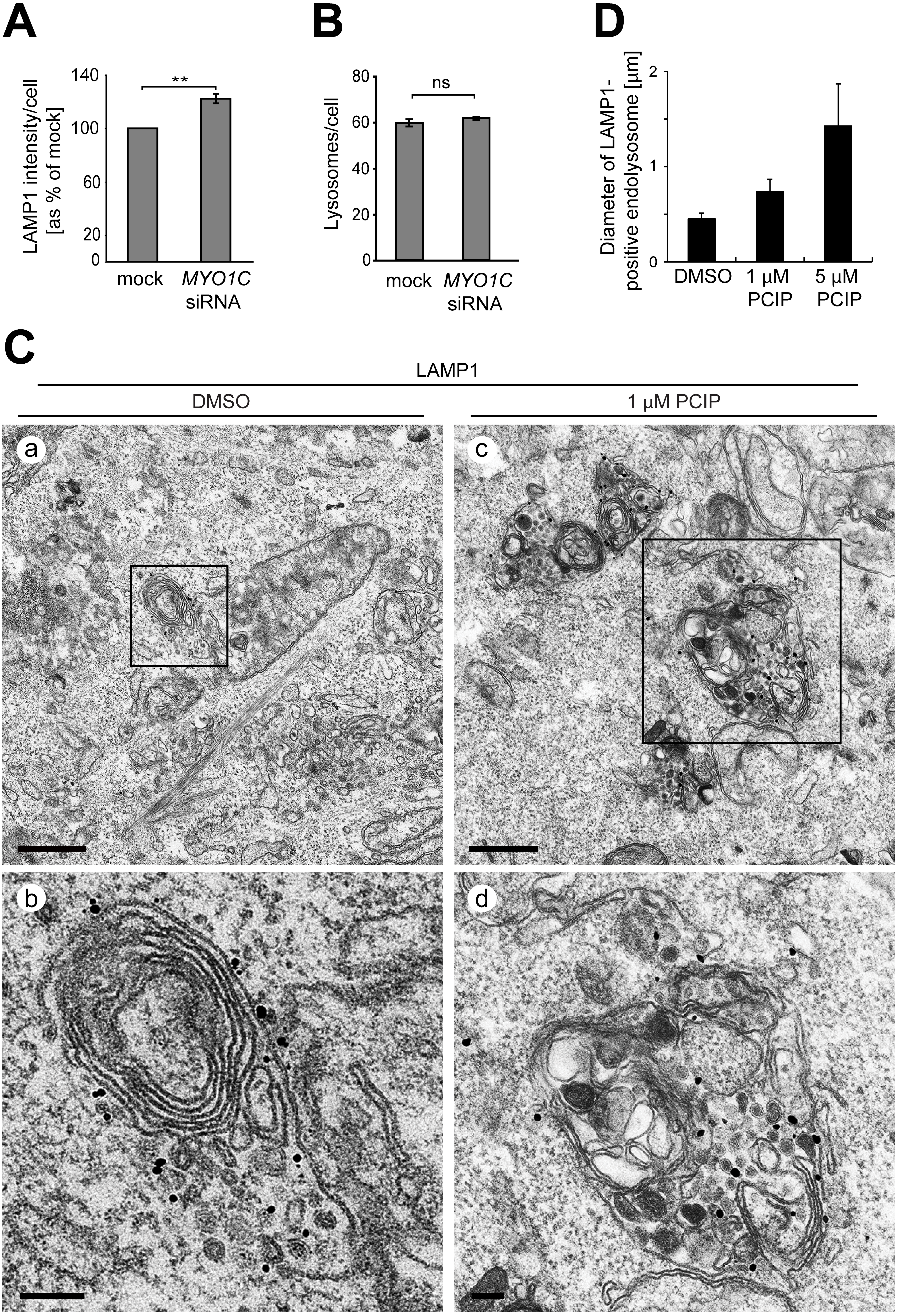

Brandstaetter et al., Supplementary figure 3
